# Plasma exchange treatment of a diabetic ketoacidosis child with hyperlipidemia to avoid pancreatitis: a case report

**DOI:** 10.3389/fped.2024.1280330

**Published:** 2024-06-05

**Authors:** Shuyue Huang, Fuying Song, Kang Gao, Yi Song, Xiaobo Chen

**Affiliations:** Department of Endocrinology, The Children’s Hospital Affiliated to the Capital Institute of Pediatrics, Beijing, China

**Keywords:** type 1 diabetes mellitus, diabetes ketoacidosis, triglycerides, pancreatitis, therapeutic intervention

## Abstract

Type 1 diabetes mellitus (T1DM) is a metabolic disorder characterized by an absolute deficiency of insulin due to pancreatic failure. Diabetes ketoacidosis (DKA) has emerged as one of the most common complications of T1DM. Although exceedingly rare, the onset of T1DM with DKA may result in lipemia secondary to severe hypertriglyceridemia (HTG), accounting for several cases in the pediatric population. Along this line, plasma exchange treatment in children with DKA and severe hyperlipidemia has only been reported in some cases. In this case report, the diagnosis of an 11-year-old girl with diabetes ketoacidosis accompanied by severe HTG, along with subsequent plasma exchange treatment, is presented. Initially, the patient received initial management with crystalloid fluid bolus and intravenous insulin therapy. Despite rapid correction of acidosis, persistent HTG subsequently prompted the plasma exchange treatment. A total of three sessions were administered over 2 days, leading to a significant reduction in the triglyceride levels and corneal opacity resolution, indicating a successful therapeutic intervention.

## Case presentation

An 11-year-old female child with no previous medical history was admitted to an emergency department after experiencing half a day of breathing difficulty and subsequent unconsciousness. During the admission, it was reported that the symptoms started 2 months prior with polydipsia and polyuria, with 3 kg weight loss. Furthermore, the patient’s condition has deteriorated by the time of admission. Prior to hospitalization, the patient had been experiencing fatigue and several symptoms of abdominal distension without pain, nausea, vomiting, or diarrhea. Importantly, there was no familial history of diabetes mellitus (DM), cholesterolemia, or hypertriglyceridemia (HTG).

After physical examination, the Glasgow coma scale of the patient was determined as E3V4M5 with a body mass index (BMI) of 14.9 kg/m^2^ (15th percentile). Notably, the patient was afebrile and normotensive, with an increased respiratory rate of 36/min, a heart rate of 140/min, and an oxygen saturation of 99% on room air. Moreover, the patient had dry skin with no signs of abnormal rashes or edema. The mucous membranes were dry, and the capillary refill was more than 5 s. Lipemia retinalis and eruptive xanthomas were absent. The chest was clear to auscultation with bilateral breath sounds. The cardiovascular examination also revealed no abnormalities, except tachycardia. Notably, the abdomen was distended, with normal bowel sounds and no pain throughout the entire abdomen. In addition, no hepatosplenomegaly or palpable masses were evident. The patient was neurologically asymptomatic with a flexible neck.

**Figure 1:**
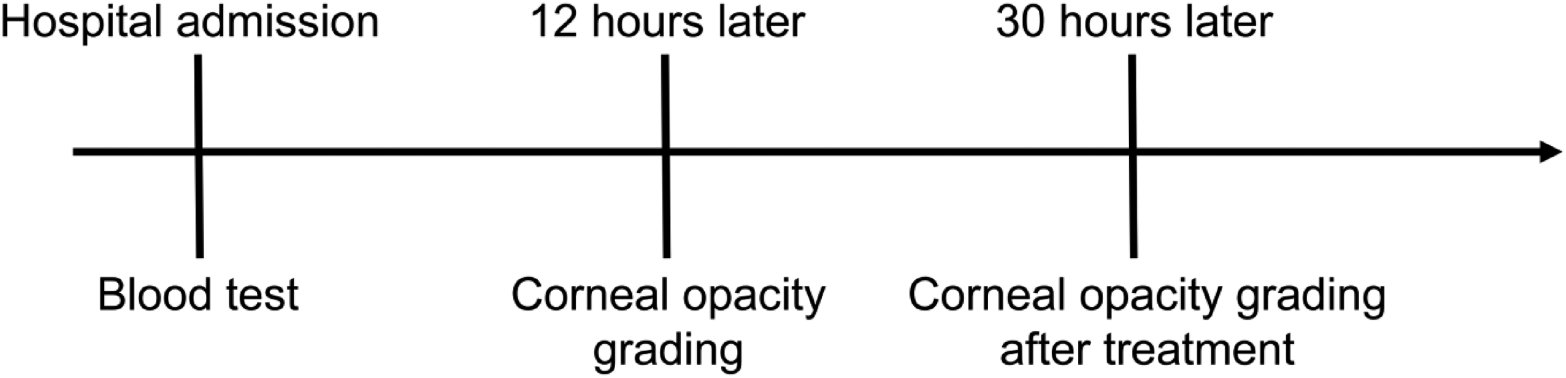
Flow chart presenting the timeline of diagnosis and treatment procedures.

**Figure 2:**
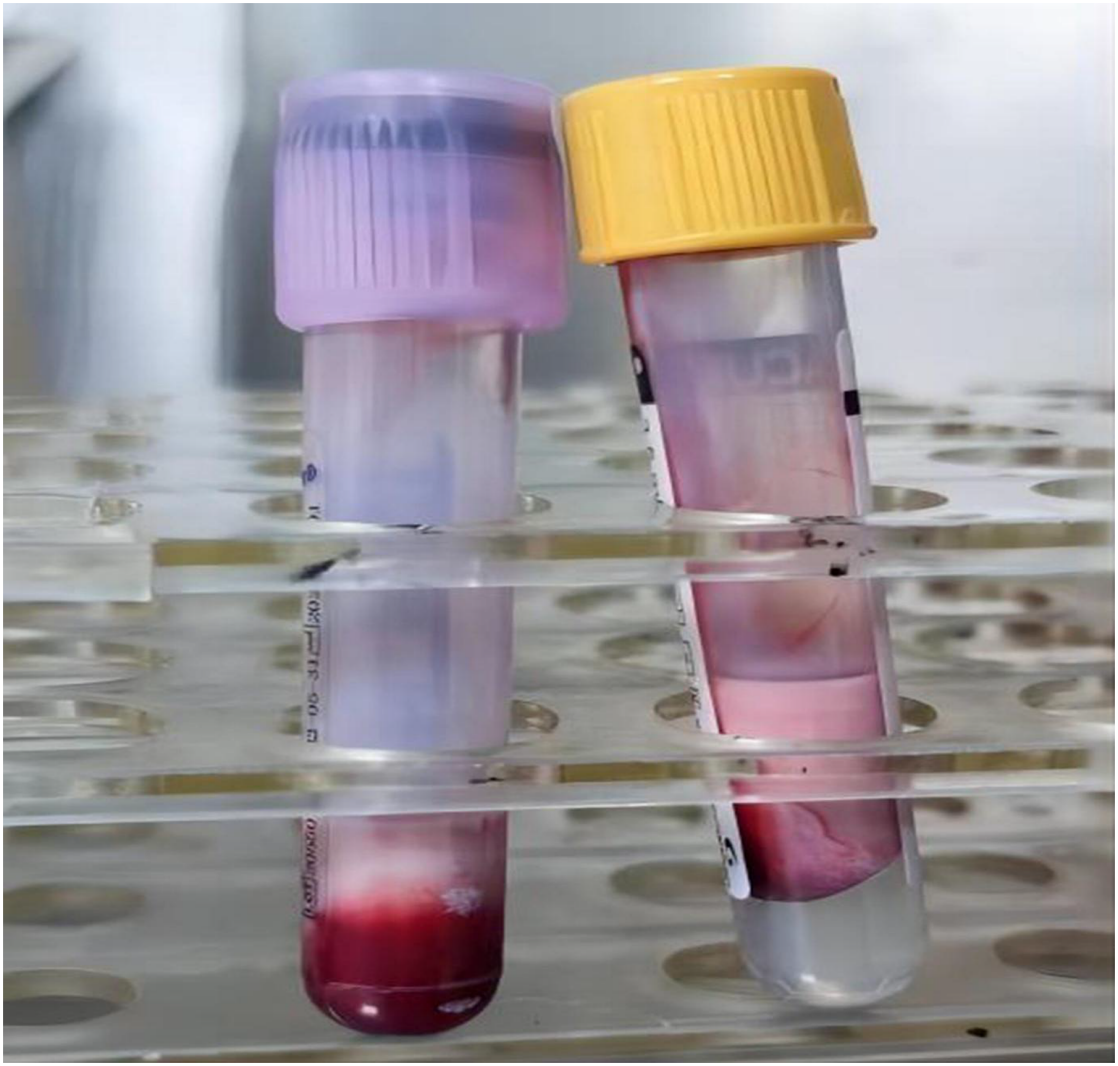
Images showing the milky blood on admission.

The timeline of diagnosis and treatment is presented in Figure 1. After admission, the collected blood for the preliminary investigations showed a milky (lipemic) appearance, indicating lipemia (Figure 2). The blood analysis indicated plasma glucose levels of 38 mmol/L (reference range of 3.9–6.1) and a β-hydroxybutyric acid level of >8.8 mmol/L (reference range of 0.03–0.3). In addition, the urine analysis was positive for urinary glucose of 4+ and urine ketones of 4+ (reference range of negative). An arterial blood gas analysis showed a pH value of 6.85 (reference range of 7.35–7.45), PCO_2_ of 28 mmHg (reference range of 35–45), HCO_3_ of 2.3 mmol/L (reference range of 22–27), a base excess of −30.3 mmol/L (reference range of ±3), and an anion gap (AG) of 23 (normal range of 12 ± 2). The serum lipid profile showed a serum triglyceride level of 22.93 mmol/L (reference range of 0–1.69) and a total cholesterol level of 6.25 mmol/L (reference range of 0–5.7). The collected blood was tested after high-speed centrifugation, resulting in a milky appearance. The serum biochemical analysis indicated sodium levels of 132.5 mmol/L (reference range of 137–145), potassium levels of 4.64 mmol/L (reference range 3.7–5.2), chloride levels of 98 mmol/L (reference range of 98–110), serum creatinine levels of 107.8 µmol/L (reference range of 27–66), blood urea nitrogen levels of 6.21 mmol/L (reference range of 2.5–6.5), serum amylase levels of 151 U/L (reference range of 25–125), urine amylase levels of 715 IU/L (reference range of <700), serum lipase levels of 214.34 U/L (reference range of 73–393), a plasma osmolality rate of 312 mOsm/kg.H_2_O, and a glycated hemoglobin (HbA1c) rate of 14.5% (reference range of 4–6). Notably, the glutamic acid decarboxylase antibody and IA-2 antibody were undetectable.

**Figure 3:**
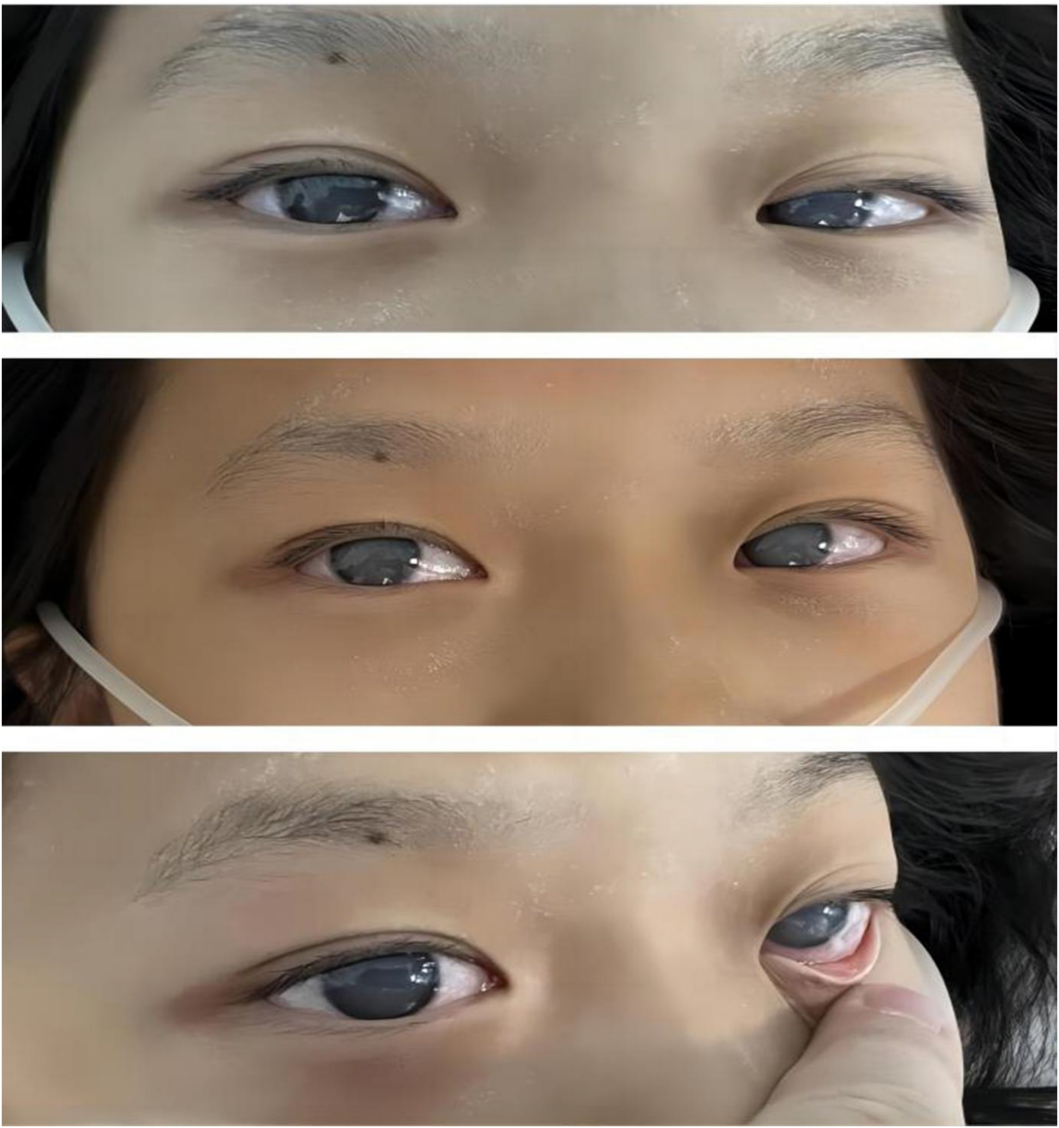
Photographic images of the rapid development of corneal opacity after admission.

After the preliminary serological investigations, the admitted patient was diagnosed with severe diabetic ketoacidosis (DKA) along with moderate-to-severe dehydration and hyperlipidemia. After the diagnosis, the patient was administered 2 L (30 ml/kg) of crystalloid fluid bolus. Despite the rapid correction of acidosis with intravenous (IV) fluids and IV insulin (0.2–0.3 U/kg.h) within several hours, the blood glucose levels of the patient dropped slowly. Nevertheless, the patient remained comatose and agitated, developing corneal opacity rapidly several hours after admission (Figure 3). In addition, the serum and urine amylase analyses indicated quickly elevated levels to 288 U/L and 1,280 IU/L, respectively.

Similarly, the serum lipase levels elevated to 670 U/L. After a multidisciplinary diagnosis, the patient was shifted to the intensive care unit (ICU) for closer monitoring due to a worsening mental status after 12 h of conventional therapy. The HTG and quickly elevated serum amylase levels indicated the probability of acute pancreatitis (AP). Thus, we decided to treat the patient by plasma exchange to decrease HTG rapidly.

A volume of 1,500 mL of plasma was exchanged, resulting in the correction of HTG by one course of plasma exchange. Moreover, the corneal opacity was relieved rapidly in a few hours, during which the patient returned to consciousness. The results showed a decrease in the HTG levels from 22.93 mmol/L to 15.1 mmol/L (Table 1), reduced hypercholesterolemia from 6.25 mmol/L to 4.1 mmol/L, and significantly decreased triglyceride levels. The second course of plasma exchange was processed in the next 2 days, resulting in the decreased levels of amylase and lipase to normal. The significant reduction in these biochemical parameters was accompanied by the intravascular volume, electrolyte replacement, and administration of IV insulin, leading to the successful reduction of DKA. The radiologic imaging studies by ultrasonography (USG) and CT of the abdomen also showed the relief of abdominal discomfort, leading to the absence of pancreatitis features.

**Table 1:** Patient's laboratory findings.

Variable	Reference range	On admission	12 h (before the first plasma exchange)	18 h (after the first plasma exchange)	42 h (after the second plasma exchange)	66 h (after the third plasma exchange)	4 days
Blood							
Urea nitrogen (mmol/L)	2.5–6.5	6.21	10.4	9.5	5.92	5.85	4.41
Creatinine (umol/L)	27–66	107.8	120	140	129.8	80	63
Sodium (mmol/L)	135–145	132.5	145	150	143	140	142
Potassium (mmol/L)	3.7–5.2	4.64	3.8	3.45	4.19	4.46	3.97
Chloride (mmol/L)	98–110	98	115	126	120	115	110
Alanine aminotransferase (U/L)	7–30	22.6	21	22	15.8	9.5	11.9
Aspartate aminotransferase (U/L)	14–44	25.7	30	46.3	41.8	15	20.4
Amylase (IU/L)	25–125	151	288	251	170	84	70
Lipase (U/L)	73–393	214.34	670	334.2	101.77	90.68	207
Glucose (mmol/L)	3.9–6.1	38	27	15	13	12	8
Triglyceride (mmol/L)	0–1.69	22.93	22.9	15.1	10.25	4.68	2.95
LDL-C (mmol/L)	1.25–3.3	0.25	0.23	0.43	0.79	1.3	2.57
HDL-C (mmol/L)	0.86–1.87	0.22	0.21	0.13	0.16	0.19	0.27
β-hydroxybutyric acid (mmol/L)	0.03–0.3	>8.8	>8.8	4.4	1.1	0.14	0.1
PH	7.35–7.45	6.85	6.99	7.167	7.316	7.425	7.36
HCO_3_- (mmol/L)	22–27	2.3	3.5	10.2	16.6	20.8	20.2
Base excess (mmol/L)	−3-+3	−30.3	−28.5	−16.7	−10.1	−6.7	−3
Urine							
Urine glucose	-	4+	4+	3+	1+	1+	-
Urine ketone	-	4+	4+	2+	1+	-	-
Urine amylase (IU/L)	<700	715	1,280	842	690	430	290

The serum lipid, β-hydroxybutyric acid, arterial blood gas analysis, and blood biochemical indices are presented in Table 1. Considering dehydration, the patient with acute renal injury presented a gradual decrease in the creatinine and urea nitrogen levels after our treatment. The patient was subsequently transferred to the general pediatric ward after 4 days of treatment in the ICU. During the treatment, the family underwent diabetic education. Eventually, the patient was discharged home on a regimen with short- and long-acting insulin. As anticipated, the patient continued to do well in the outpatient follow-up, maintaining good glycemic control and normal triglyceride levels.

## Discussion

Emerging as one of the most recurrent problems in the emergency room, DKA requires prompt and effective treatment. Notably, hyperlipidemia is often observed during DKA and is represented as HTG (1). The key pathophysiological cause of DKA is the lack of insulin. To this end, lipoprotein lipase (LPL), which is the enzyme responsible for TG metabolism, converts very low-density lipoprotein (VLDL) and chylomicrons (CL) into free fatty acids (FFA). Accordingly, the absence of insulin in the blood results in the accumulation of the aforementioned TG-rich lipoproteins (2). Thus, the LPL enzyme is unable to effectively metabolize the VLDL and CL into FFA in the blood. The insulin-deficient state, such as in DKA, results in the propensity for HTG levels (3). Consequently, the marked elevation of serum triglycerides occurs during episodes of DKA (4). Although the HTG rarely attains the noted levels, severe HTG may increase the risk of AP in DKA patients (5). According to the Guidelines of the Endocrine Society, severe HTG is referred to as a triglyceride level of >1,000 mg/dL (11.3 mmol/L), while very severe HTG is referred to as a triglyceride level of >2,000 mg/dL (22.6 mmol/L). Accordingly, severe HTG has only been recorded in 8% of the adult population with DKA, with limited documented cases in the pediatric population (6). However, triglyceride levels of >1,000 mg/dL (11.3 mmol/L) could reportedly cause a 5% risk for the development of AP, which could be increased to 10%–20%, with triglyceride levels of >2,000 mg/dL (22.6 mmol/L) (7). The triad phenomenon of DKA, AP, and severe HTG is rare, particularly in children (8). Although the pathogenesis of AP in DKA varies, some transient and profound hyperlipidemia is an identifiable factor. Pancreatitis is caused by the breakdown of triglycerides by pancreatic lipase into FFA, which become toxic to the pancreas (9).

To this end, plasma exchange therapy was first reported in 1975 to treat familial hypercholesterolemia (10), adult hyperlipidemia, and DKA with lesional pulmonary edema and in 1982 to treat AP (11). Despite its success in adults, there are very few available reports on the use of plasma exchange in children. In 2012, Lutfi et al. (12) reported the first pediatric case of DKA, AP, and severe HTG successfully treated with plasmapheresis. Previous reports indicated that the plasma triglyceride levels could be reduced to less than 500 mg/dL within 3–17 days in most cases by conventional treatment (6). After admission, the patient with DKA and severe HTG was unresponsive to conventional treatment by IV fluid and IV insulin. In this context, the blood amylase, urine amylase, and blood lipase were elevated rapidly. If severe hyperlipidemia is not corrected, it will soon lead to a life-threatening AP. Accordingly, we decisively implemented the plasma exchange treatment, consequently resulting in a positive effect on the dramatic reduction of triglycerides. After three times of plasma exchange, the blood amylase, urine amylase, and blood lipase levels were decreased to nearly normal, reducing the risk of life-threatening pancreatitis. These results were in agreement with the reported literature indicating the efficacy of plasma exchange in patients with DKA.

**Figure 4:**
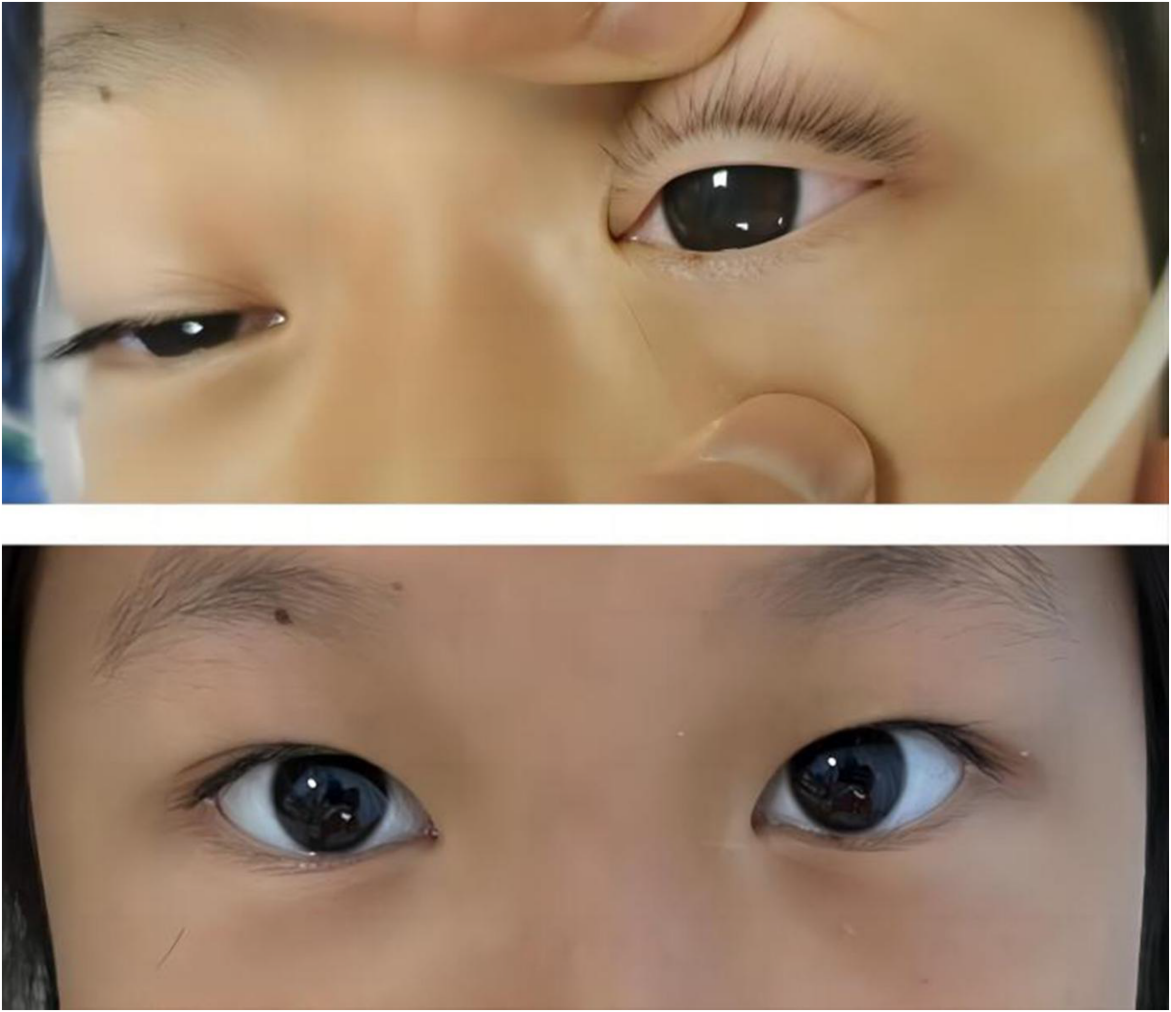
Photographic images of the relieved corneal opacity after several hours of the first plasma exchange treatment.

**Figure 5:**
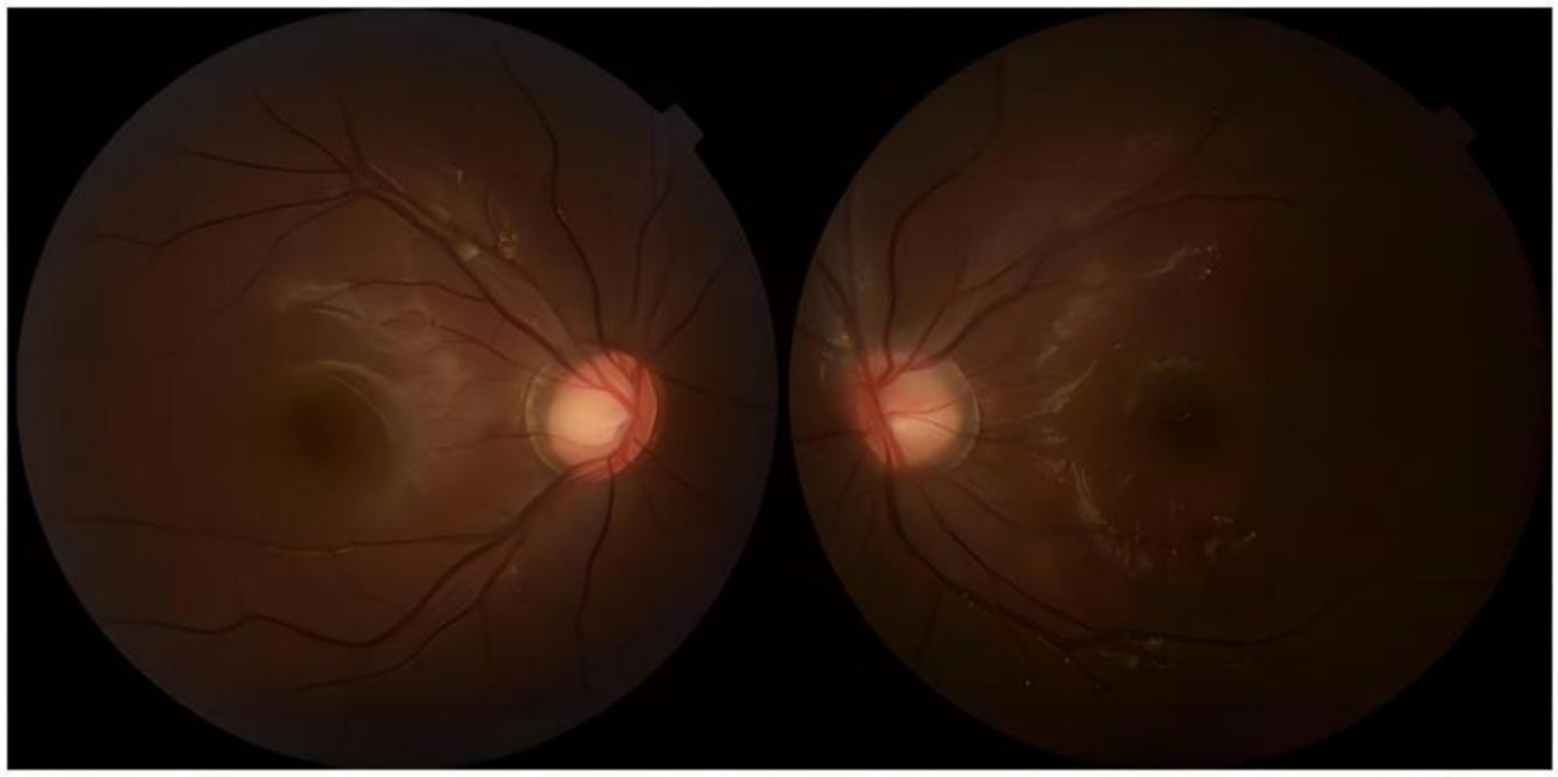
Image showing the fundoscopic examination.

Notably, patients with severe HTG are at risk of the additional comorbidities of lipemia retinalis. This complication can be observed in up to 23% of patients with severe HTG (>2,000 mg/dL) (13). Similarly, the patient described in this case report developed progressive corneal opacity. However, this corneal opacity rapidly recovered during the first plasma exchange (Figure 4). Accordingly, the ophthalmologist assumed that this performance was not retinal lipidemia due to the lack of obvious abnormalities in later fundoscopic examinations (Figure 5). Furthermore, the blood lipid level was close to normal, and the patient recovered from DKA. To the best of our knowledge, no similar performance was reported. The occurrence and remission of corneal opacity could be directly related to the triglyceride levels, which might be due to the deposition of lipids on the cornea.

It should be noted that all the diagnosed analyses resulted in recorded TG values of >1,000 mg/dL, lending credence to the theory of a risk factor for AP. Notably, most of the individuals with higher TG levels may experience more chances of AP. Contrarily, a pediatric child with an elevated TG level (14,461 mg/dL) showed no symptoms of AP (14). The mechanism of HTG in DKA was postulated to entail an increased release of FFA from adipocytes through stimulation by counterregulatory hormones, leading to a reduction in the VLDL-TG clearance (15). Nonetheless, pathophysiology in the cases of severe HTG could not be fully attributed to insulin insufficiency alone. Reasonably, a concomitant genetic predisposition to HTG, particularly mutations in the chromosome 8 gene encoding for LPL, might exacerbate the condition. In this vein, mutations (>100) causing autosomal recessive inheritance have been documented in the literature for the corresponding LPL encoding gene (16).

## Patient’s perspective

Eventually, the patient reported that she felt markedly better after the plasma exchange treatment, with no symptoms of fatigue, abdominal distension, vomiting, or diarrhea.

## Conclusion

In summary, our case reported a rare occurrence of severe HTG with DKA in pediatric children. Although severe HTG is seldom observed in the pediatric population, keeping HTG in mind in pediatric patients with DKA presentation could be important as it might guide further clinical treatment. Notably, plasma exchange should be attempted in cases of decompensated diabetes associated with severe HTG, which could rapidly and effectively reduce the level of blood triglyceride and prevent the occurrence of severe complications, such as AP. In this single-case report, we believe that the findings may not be generalized to all pediatric patients with DKA and severe HTG. Although the immediate response to the treatment was well-documented, long-term follow-up data on the patient's glycemic control, lipid profile, and overall health outcomes remained unexplored. Although the plasma exchange is a resource-intensive procedure, this procedure may not be readily available in all healthcare settings, limiting its widespread applicability.

## Data Availability

The raw data supporting the conclusions of this article will be made available by the authors, without undue reservation.
